# Vibroacoustic lung therapy and early postoperative oxygenation after cardiac surgery: a multicentre prospective study

**DOI:** 10.3389/fmed.2026.1945462

**Published:** 2026-09-17

**Authors:** Timur Kapyshev, Marat Shoranov, Zhangeldi Kadirov, Anton Zatsarinnyy, Daniyar Arenov, Seilkhan Zhunisov, Mikhail Vorontsov, Ildar Fakhradiyev

**Affiliations:** 1 National Scientific Cardiac Surgery Center, Astana, Kazakhstan; 2 S.D. Asfendiyarov Kazakh National Medical University, Almaty, Kazakhstan; 3 Atyrau Regional Cardiology Center, Atyrau, Kazakhstan; 4 City Cardiology Center, Almaty, Kazakhstan; 5 Regional Cardiology Center, Uralsk, Kazakhstan; 6 Heart Center Shymkent, Shymkent, Kazakhstan; 7 Research Institute of Cardiology and Internal Diseases, Almaty, Kazakhstan; 8 College of Medicine, Korea University, Seoul, Republic of Korea

**Keywords:** cardiac surgery, FiO_2_, oxygenation, P/F ratio, postoperative respiratory failure, vibroacoustic therapy

## Abstract

**Background:**

Postoperative respiratory dysfunction remains a clinically significant problem after cardiac surgery. However, clinical evidence on vibroacoustic pulmonary therapy in adult cardiac surgery patients remains limited. The study aimed to compare early postoperative oxygenation following vibroacoustic pulmonary therapy using VibroLUNG with routine manual chest physiotherapy after cardiac surgery.

**Methods:**

This prospective, multicentre, observational, non-randomized comparative study included adult patients aged 18–80 years who underwent elective cardiac surgery and developed postoperative respiratory dysfunction meeting the study eligibility criteria. Group allocation reflected routine clinical practice and device availability at the participating centers. Patients received either vibroacoustic pulmonary therapy using VibroLUNG or routine manual percussion chest physiotherapy. The principal respiratory outcome analysed in this manuscript was the P/F ratio at the early postoperative assessment. Key secondary outcomes included FiO₂ requirement, SpO₂, PaO₂, PaCO₂, acid–base status parameters, clinical scales, radiographic findings, length of stay, and recorded safety outcomes.

**Results:**

Of 354 screened patients, 317 were included in the analysis: 156 in the VibroLUNG group and 161 in the control group. Baseline anthropometric characteristics were comparable; however, the distribution by center/region differed substantially between the groups. At the early postoperative assessment, the VibroLUNG group had a lower administered FiO₂ [35.0% [30.0–41.0] vs. 41.5% [40.0–50.0], *p* < 0.001]. In the principal centre-adjusted analysis, the P/F ratio was higher in the VibroLUNG group by 23.8 units (95% CI 8.1–39.5; *p*=0.003). Before extubation, FiO₂ remained lower in the VibroLUNG group [30.0% [30.0–35.0] vs. 35.0% [30.0–50.0], *p* < 0.001], whereas the unadjusted difference in the P/F ratio was not statistically significant. No device-related adverse events, device deficiencies, or deaths during study participation were recorded.

**Conclusion:**

VibroLUNG use was associated with a higher early postoperative P/F ratio and lower administered FiO₂. No adverse events, device deficiencies, or deaths were recorded during the study observation period. Causal inference is limited by the non-randomized design and centre-level differences.

## 1 Introduction

Respiratory dysfunction is common after cardiac surgical procedures and may be associated with general anaesthesia, cardiopulmonary bypass, atelectasis, and other perioperative factors (1). Postoperative pain may further interfere with effective breathing and respiratory exercises (2, 3). Postoperative respiratory failure may delay extubation and increase oxygen-support requirements (4, 5).

Chest physiotherapy is commonly used as part of postoperative respiratory care, including after thoracic and cardiac surgery (6, 7). Common approaches include positioning, postural drainage, vibration, breathing exercises, positive expiratory pressure techniques, and other airway-clearance methods (6). Deep-breathing exercises after cardiac surgery have been associated with reduced atelectasis and improved pulmonary function (7), while high-frequency chest wall oscillation has been evaluated as an adjunctive respiratory therapy in postoperative thoracic surgery (8, 9).

However, the available evidence remains heterogeneous with respect to patient populations, intervention protocols, and measured outcomes (10, 11). These limitations support the evaluation of newer non-invasive respiratory physiotherapy technologies in postoperative care.

BARK VibroLUNG is a registered medical device for chest physiotherapy that delivers variable-frequency vibroacoustic stimulation through two external emitters applied to the chest wall, with a frequency range of 20–300 Hz.

Although vibration- and oscillation-based airway-clearance approaches have been investigated in postoperative care (8–11), multicentre comparative data on vibroacoustic pulmonary therapy in adult cardiac surgery patients remain limited, particularly regarding early postoperative oxygenation and oxygen-support requirements.

The study aimed to compare early postoperative oxygenation following vibroacoustic pulmonary therapy using BARK VibroLUNG with routine manual percussion chest physiotherapy after cardiac surgery.

## 2 Materials and methods

### 2.1 Study design and setting

This was a prospective, multicentre, post-registration, non-interventional, observational, non-randomized comparative study of BARK VibroLUNG in adult cardiac surgery patients. The study was registered on ClinicalTrials.gov (NCT06320483). The first patient first visit took place on 20 September 2023, and the last patient last visit took place on 21 March 2025. The study was conducted in 11 cardiac surgery or cardiology centres in Kazakhstan. Nine of the 11 participating centres contributed patients to the analysed cohort. Treatment allocation was non-randomized and was primarily determined by centre-level availability of the VibroLUNG device. At the beginning of the study, participating centres were effectively grouped according to whether the device was available in routine practice. During the study, treatment availability changed at some centres, and control recruitment was subsequently opened across participating sites. Consequently, both treatment groups were represented within several analysed site/region strata, although treatment overlap remained incomplete.

### 2.2 Device description and principle of action

BARK VibroLUNG is a registered medical device in the Republic of Kazakhstan [registration certificate RK-MD (MT) No. 013611]. The device is intended for chest physiotherapy in medical organizations and may be used by trained physicians or nursing staff. The system consists of a base unit and two handheld vibroacoustic emitters connected to the unit by a twisted cable. The touchscreen display allows the operator to select the clinical profile/programme, age category, and body type of the patient before the procedure.

The physical principle is based on the external transmission of variable-frequency sound waves and vibration through the chest wall (floating frequency range 20–300 Hz). The hypothesized physiological effects include mechanical support of airway clearance and ventilation through mobilization of bronchial secretions, reduction of small-airway obstruction, recruitment of hypoventilated alveolar units, and improvement of ventilation–perfusion matching (Figure 1).

**Figure 1:**
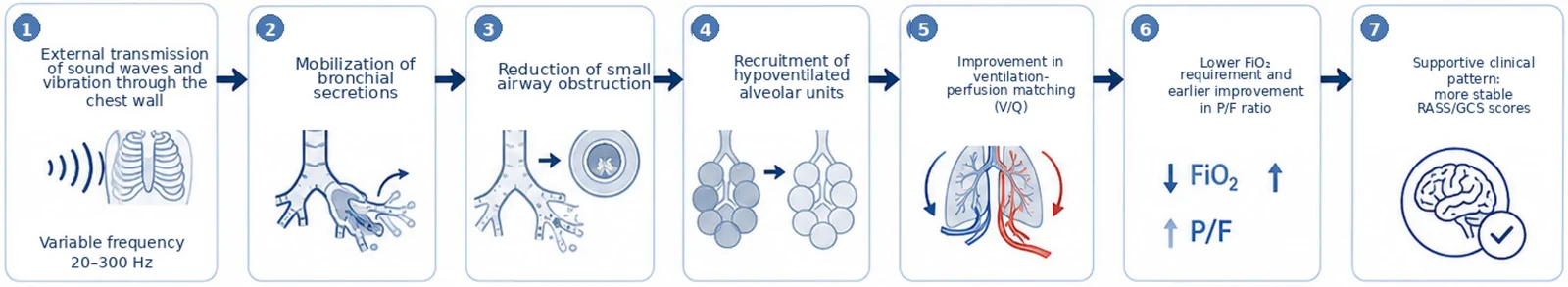
Hypothesized physiological mechanisms of VibroLUNG in early postoperative respiratory care.

The procedure is performed for 5 min. After self-testing and selection of the appropriate programme, the emitters were applied to the chest. The emitters were automatically activated upon contact with the surface and deactivated or reactivated when the position was changed. After completion of the procedure, the device switched to stop mode. The device has absolute and relative contraindications, including unstable clinical condition, shock, high risk of bleeding in the exposure area, unstable rib fracture, risk of pneumothorax or haemothorax, and the presence of implanted electromechanical devices in the projection of exposure.

### 2.3 Study participants

Eligible participants were men and women aged 18–80 years who were hospitalized for elective cardiac surgery, including coronary artery bypass grafting, internal mammary artery bypass grafting, valve repair or replacement, and repair of an atrial or ventricular septal defect.

The inclusion criteria were a body mass index of 18.5–35 kg/m², ASA physical status II–III, general anaesthesia with tracheal intubation and mechanical ventilation lasting more than 3 h, cardiopulmonary bypass lasting no more than 150 min, a preoperative left ventricular ejection fraction greater than 40%, and postoperative respiratory dysfunction meeting the study eligibility criteria.

Postoperative respiratory eligibility was established during the early ICU period and required a P/F ratio >150 together with clinical stability, including stable haemodynamics with limited vasoactive support, a Glasgow Coma Scale score >12, absence of acute kidney injury, critical coagulopathy or acute liver failure, and adequately controlled pain and delirium.

The main exclusion criteria included implanted or external electromechanical devices within the treatment area, severe hypocoagulation or hypercoagulation, a recent acute cerebrovascular event, unstable rib fracture, risk of pneumothorax or haemothorax, severe bullous lung disease, severe kyphoscoliosis, and an estimated systolic pulmonary artery pressure greater than 50 mmHg. Mechanical heart valves and other major endothoracic or endovascular implants were considered relative contraindications and were taken into account during eligibility assessment.

### 2.4 Intervention and comparator

Patients in the intervention group received vibroacoustic pulmonary therapy using BARK VibroLUNG as part of standard postoperative care during the early postoperative ICU period. Each session was performed using the “intensive care: prevention” programme for 5 min and was repeated three times daily for 3 postoperative days. The procedures were performed by physicians or nursing staff who had received specific instruction in the use of the device. Patient positioning was determined by the postoperative clinical condition; no specific body position was mandated by the VibroLUNG protocol. Two vibroacoustic emitters were applied to the chest wall according to the device-use recommendations and could be repositioned during the procedure as required.

Patients in the control group received routine manual percussion chest physiotherapy according to local clinical practice and the rehabilitation protocols of the participating centres, including the Clinical Protocol for Medical Rehabilitation “Second Stage “Rehabilitation II A”, Profile “Cardiology and Cardiac Surgery” (Adults)” (Kazakhstan, 2014). The duration, frequency, and specific anatomical technique of manual percussion were not mandated by a uniform study protocol and reflected routine local practice. Manual percussion was delivered by clinical staff involved in routine postoperative rehabilitation.

As an adjunct to postoperative respiratory rehabilitation, patients performed breathing exercises using incentive spirometry three times daily. Other postoperative management, including oxygen therapy, ventilatory support, analgesia, and concomitant treatment, was determined by the attending physicians according to the patient's clinical condition and national and local standards. Because the study was non-interventional, no study-specific protocol mandated a particular oxygen-delivery device, oxygen flow rate, ventilator setting, PEEP level, extubation criterion, or oxygen-titration target.

Study assessments were linked to clinically defined perioperative milestones rather than fixed elapsed times after surgery. The early postoperative assessment represented the initial postoperative ICU assessment, the pre-extubation assessment was performed before removal of the endotracheal tube, and the post-overnight assessment was performed after the subsequent overnight recovery period. Exact intervals from surgery and from individual treatment sessions were not uniformly fixed across centres; therefore, these assessments were not interpreted as standardized immediate pre- and post-treatment measurements.

Although eligibility was established postoperatively, the study did not mandate a standardized respiratory assessment immediately before the first VibroLUNG or manual-percussion session. Therefore, the preoperative P/F ratio was used only as a baseline covariate and was not considered a surrogate for respiratory status at treatment initiation.

Because VibroLUNG treatment and manual percussion were visibly different procedures, treating personnel were not blinded to treatment allocation. Outcome data were obtained from routine clinical and physiological assessments, and no separate blinded central outcome-adjudication procedure was incorporated into the study design.

### 2.5 Endpoints

The present manuscript focuses on the P/F oxygenation ratio at the early postoperative assessment as the principal respiratory outcome. The registered study included multiple primary respiratory outcomes; therefore, the P/F ratio is presented here as the principal respiratory outcome of this focused manuscript rather than as the sole protocol-defined primary endpoint. The P/F ratio was calculated as the ratio of PaO₂ to the FiO₂ documented at the corresponding arterial blood-gas assessment, with FiO₂ expressed as a fraction in the calculation.

Key secondary respiratory outcomes included FiO₂, SpO₂, PaO₂, and PaCO₂. FiO₂ was assessed during the early postoperative period and before extubation. SpO₂ was interpreted together with the level of supplemental oxygen administered and was not considered independently of FiO₂.

Acid–base parameters, including pH and base excess (BE), as well as the RASS (12), Glasgow Coma Scale (13), BPS (14), NRS (15), and radiographic findings, were considered supportive or exploratory outcomes. The category “other clinically significant radiographic changes” included nonspecific abnormalities such as hypoventilated areas, subsegmental collapse, secretion stasis, peribronchial changes, and early disatelectatic changes. No central blinded radiographic adjudication was incorporated into the study design. These measures were interpreted in the context of perioperative factors, including sedation, analgesia, extubation status, and baseline radiographic findings.

Haematological and coagulation parameters, including complete blood count, prothrombin time, international normalized ratio (INR), fibrinogen, and activated partial thromboplastin time (aPTT), were considered exploratory and hypothesis-generating outcomes and were not used to assess treatment efficacy.

Safety surveillance was conducted throughout study participation by the treating clinical teams and included recording of adverse events, device deficiencies, technical problems, and deaths in accordance with applicable clinical safety-reporting procedures. The possible relationship of reported events to the device or procedure was assessed where applicable. The observation period covered the early postoperative ICU period and subsequent follow-up for up to 6 days in the specialty ward. No independent central safety adjudication was incorporated into the study design.

The ROX index was analysed only in patients for whom SpO₂, FiO₂, and respiratory rate were simultaneously available at the corresponding assessment time point. If any of these components was missing, the index was not calculated.

### 2.6 Statistical analysis

Continuous variables are presented as median (interquartile range), and categorical variables as n (%). Distributional assumptions were assessed using the Shapiro–Wilk test. Because most continuous variables showed non-normal distributions, between-group comparisons were performed using the Mann–Whitney U test, while paired within-group comparisons over time were performed using the Wilcoxon signed-rank test. Categorical variables were compared using the *χ*² test or Fisher's exact test, as appropriate. Rank-biserial correlation was calculated as an effect-size measure for non-parametric comparisons. Standardized mean differences (SMDs) were additionally calculated to assess between-group balance in baseline characteristics.

For outcomes measured repeatedly across study visits and showing non-normal distributions, aligned rank transform analysis was used to evaluate the main effects of group and time and the group-by-time interaction. When statistically significant interactions were identified, *post-hoc* pairwise comparisons were performed with Holm–Bonferroni adjustment for multiple testing. Effect estimates are reported with 95% confidence intervals where applicable.

The ART analyses accounted for the repeated-measures structure over time but did not incorporate study centre as a multilevel effect. Centre-level confounding was addressed separately for the principal early postoperative P/F-ratio analysis through adjustment for study centre and through the sensitivity analysis restricted to site/region strata in which both treatment groups were represented.

Holm–Bonferroni correction was applied to *post-hoc* pairwise comparisons following significant ART interactions. No global multiplicity adjustment was applied across all exploratory outcome domains; therefore, physiological, clinical, laboratory, and radiographic analyses other than the principal respiratory outcome were interpreted as supportive or exploratory rather than confirmatory.

For the early postoperative P/F-ratio outcome, the principal analysis estimated the between-group difference after adjustment for study centre, with centre included as an adjustment factor. A sensitivity analysis additionally included preoperative P/F ratio, while a separate sensitivity analysis was restricted to site/region strata in which both treatment groups were represented. Adjusted between-group differences are reported with 95% confidence intervals and *p* values, whereas the unadjusted comparison is presented descriptively.

Permutation-based ANCOVA was used as a supportive analysis of the P/F ratio to reduce dependence on conventional parametric distributional assumptions. PaO₂ and FiO₂ were included as covariates, and the permutation analysis was implemented in R using the lmPerm package. Length of stay was analysed using ordinal logistic regression (proportional odds model), with treatment group and region included in the model; results are reported as odds ratios with 95% confidence intervals.

Analyses were performed using Jamovi version 2.4.8, IBM SPSS Statistics version 19, and R version 4.4.3. R packages used for statistical computation and data presentation included rstatix, ARTool, emmeans, dplyr, flextable, and lmPerm. Statistical significance was defined as a two-sided *p* < 0.05.

Analyses were based on available observations for each outcome and assessment time point; no imputation of missing data was performed. Outcome-specific denominators and missing observations for key respiratory measurements are reported in Supplementary Table S3. Given the non-randomized design, potential confounding related to study centre, treatment availability, oxygen-management practices, extubation criteria, sedation, analgesia, and completeness of documentation was considered when interpreting the findings.

The original sample-size calculation was based on the expected difference in the proportion of patients achieving normal oxygen saturation. Assuming effectiveness rates of 86% in the percussion-massage group and 96% in the vibroacoustic-therapy group, a two-sided significance level of 0.05, 80% power, and a 1:1 group ratio, 129 participants per group were required. After adding a 20% allowance for attrition, the planned sample size was 155 participants per group, corresponding to a total target enrolment of 310 participants. The final analysed cohort comprised 317 participants, slightly exceeding the planned target of 310.

## 3 Results

### 3.1 Study population and baseline characteristics

A total of 354 patients underwent screening, of whom 317 were included in the analysis (156 in the VibroLUNG group and 161 in the control group). Of the 37 screened patients who were not included, 24 did not meet the postoperative eligibility criteria, while 13 were not enrolled because recruitment to the VibroLUNG group had already been completed.

Baseline clinical and perioperative characteristics are summarized in Table 1. The largest between-group imbalance was observed for other significant preoperative radiographic findings (SMD=0.76). Moderate imbalances were also present in preoperative P/F ratio (SMD=0.35), PaO₂ (SMD=0.30), and PaCO₂ (SMD = 0.25), with smaller imbalances in several other baseline and perioperative characteristics.

**Table 1:** Baseline clinical and perioperative characteristics.

Characteristic	VibroLUNG (*n* = 156)	Control (*n* = 161)	SMD
Age, years, median [IQR]	62 [56–67]	63 [57–68]	0.10
Male sex, *n* (%)	119 (76.3%)	112 (69.6%)	0.15
BMI, kg/m², median [IQR]	27.93 [25.47–31.12]	28.41 [25.06–31.22]	0.10
Coronary artery disease, *n* (%)	143 (91.7%)	138 (85.7%)	0.19
Type of cardiac surgery			
CABG/IMA bypass, *n* (%)	140 (89.7%)	135 (83.9%)	0.17
Valve repair/replacement, *n* (%)	13 (8.3%)	22 (13.7%)	0.17
Other cardiac surgery, *n* (%)	3 (1.9%)	4 (2.5%)	0.04
ASA physical status			
ASA II, *n* (%)	54 (34.6%)	51 (31.7%)	0.06
ASA III, *n* (%)	102 (65.4%)	110 (68.3%)	0.06
Smoking history, current/former, *n* (%)	63 (40.4%)	70 (43.5%)	0.06
Pre-existing pulmonary disease, *n* (%)	16 (10.3%)	20 (12.4%)	0.07
Preoperative P/F ratio, median [IQR]	391.43 [361.90–433.33]	411.43 [361.90–433.33]	0.35
Preoperative PaO₂, mmHg, median [IQR]	82.40 [76.00–91.60]	86.40 [76.50–91.00]	0.30
Preoperative PaCO₂, mmHg, median [IQR]	37.10 [34.70–39.00]	36.00 [32.30–38.00]	0.25
Preoperative FiO₂, %, median [IQR]	21 [21–21]	21 [21–21]	0.00
Cardiopulmonary bypass duration, min, median [IQR]	92 [76–111]	95 [78–114]	0.10
Aortic cross-clamp duration, min, median [IQR]	55 [42–69]	58 [44–72]	0.12
Mechanical ventilation duration, h, median [IQR]	6.1 [5.0–7.0]	6.3 [5.2–7.2]	0.10
Postoperative vasoactive support, *n* (%)	71 (45.5%)	80 (49.7%)	0.08
Blood transfusion, *n* (%)	29 (18.6%)	34 (21.1%)	0.06

Treatment distribution differed substantially across study sites. Across the 9 site/region strata represented in the analysed dataset, 5 included patients from both treatment groups, one included only VibroLUNG-treated patients, and three included only control patients (Table 2). Before surgery, the median P/F ratio was 391.43 [361.9–433.3] in the VibroLUNG group and 411.4 [361.90–433.3] in the control group (*p* = 0.253; Figure 2). Despite the non-significant conventional between-group comparison, the SMD of 0.35 indicated baseline imbalance in P/F ratio. Preoperatively, median FiO₂ was 21% in both groups (Figure 3).

**Table 2:** Distribution of participants by analysed site/region stratum and treatment group.

Site/region code	VibroLUNG, *n* (%)	Control, *n* (%)	Total, n
Region 1	40 (25.6%)	7 (4.3%)	47
Region 2	5 (3.2%)	16 (9.9%)	21
Region 6	10 (6.4%)	23 (14.3%)	33
Region 7	80 (51.3%)	22 (13.7%)	102
Region 11	5 (3.2%)	0 (0%)	5
Region 16	16 (10.3%)	3 (1.9%)	19
Region 17	0 (0%)	41 (25.5%)	41
Region22	0 (0%)	15 (9.3%)	15
Region32	0 (0%)	34 (21.1%)	34
Total enrolled	156 (100%)	161 (100%)	317

**Figure 2:**
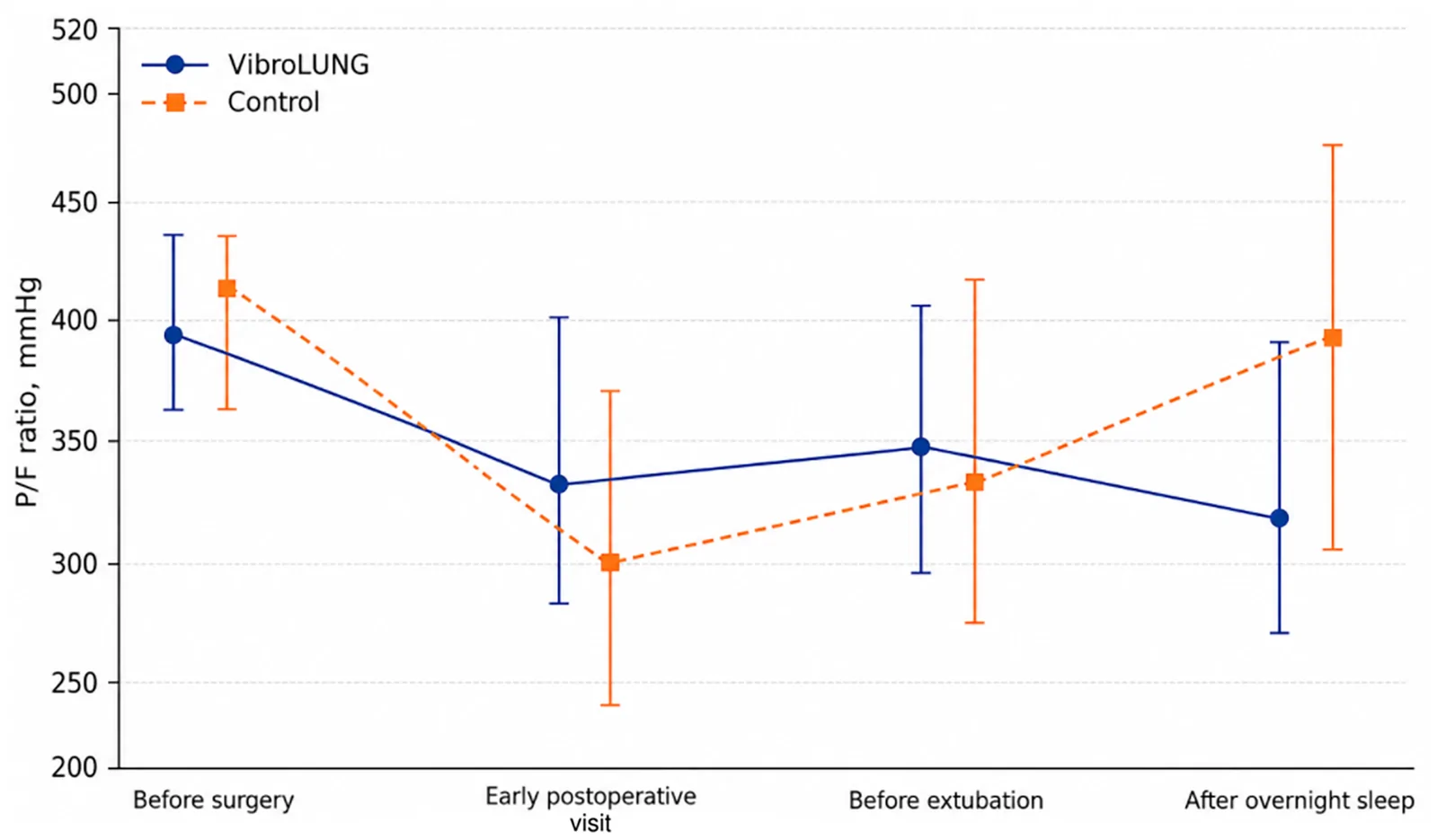
Median P/F ratio by clinical assessment milestone. Error bars represent the interquartile range (IQR). Available observations (VibroLUNG/control) were *n* = 118/75 before surgery, *n* = 151/143 at the early postoperative assessment, *n* = 144/150 before extubation, and *n* = 154/143 after overnight sleep. Assessments were defined by clinical milestones, and exact elapsed times from surgery were not uniformly standardized across centres.

**Figure 3:**
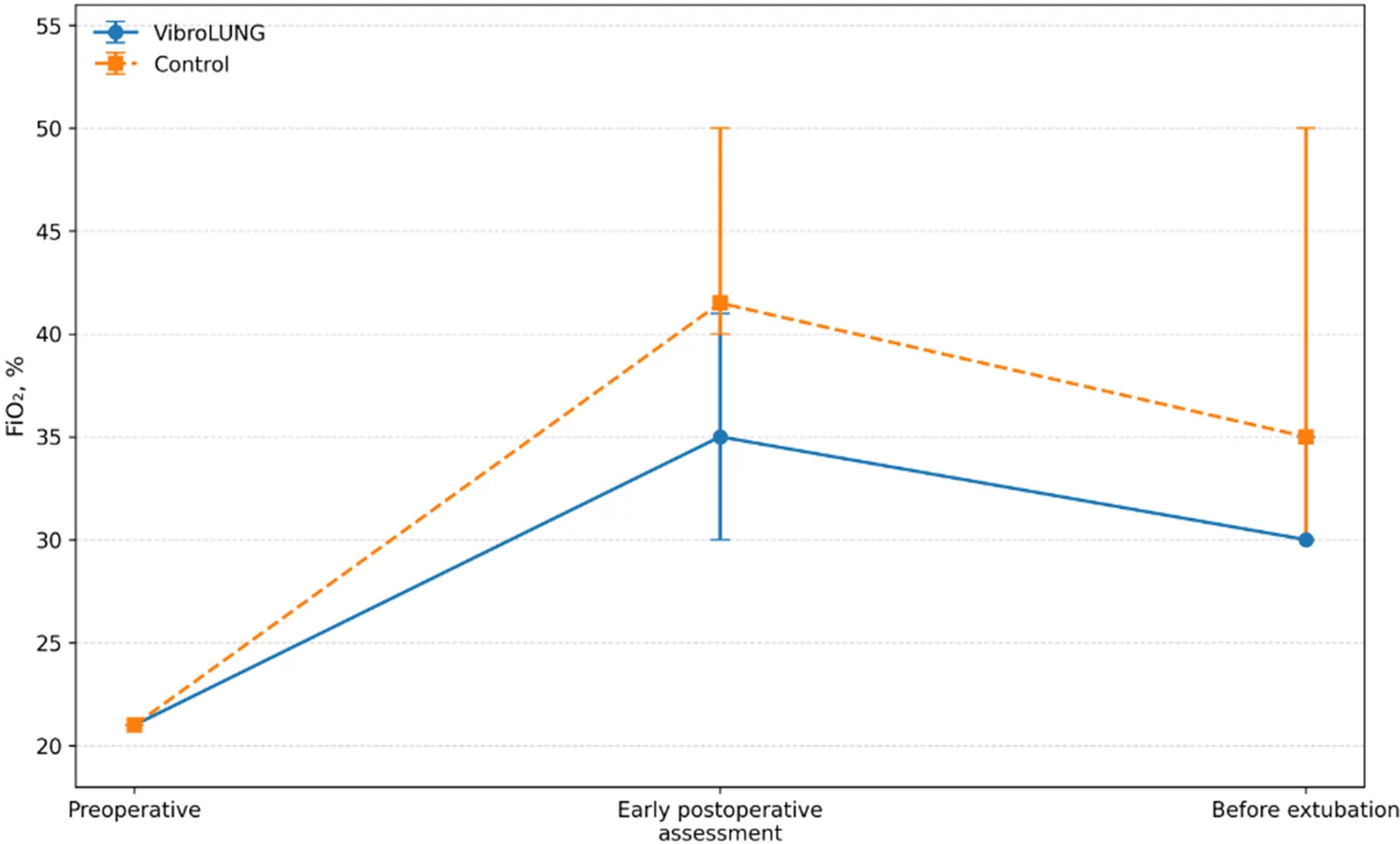
Median FiO₂ by clinical assessment milestone. Error bars represent the interquartile range (IQR). Available observations (VibroLUNG/control) were *n* = 127/108 preoperatively, *n* = 153/154 at the early postoperative assessment, and *n* = 156/157 before extubation. Assessments were defined by clinical milestones, and exact elapsed times from surgery were not uniformly standardized across centres.

At the early postoperative assessment, the VibroLUNG group had a lower administered FiO₂ [35.0% [30.0–41.0] vs. 41.5% [40.0–50.0], *p* < 0.001; Figure 3] and a higher unadjusted P/F ratio [333.3 [284.0–401.6] vs. 302.5 [240.0–370.0], *p* < 0.001; Figure 2]. In the centre-adjusted analysis, the estimated between-group difference in P/F ratio was +23.8 units (95% CI +8.1–+39.5; *p* = 0.003). The association remained statistically significant after additional adjustment for preoperative P/F (+18.9 units; 95% CI +1.9–+35.9; *p* = 0.030) and in the analysis restricted to site/region strata in which both treatment groups were represented (+20.7 units; 95% CI +3.4–+38.0; *p* = 0.019) (Table 3).

**Table 3:** Early postoperative P/F-ratio outcome: unadjusted, adjusted, and sensitivity analyses.

Analysis	VibroLUNG/Control, *n*	VibroLUNG P/F, median [IQR]	Control P/F, median [IQR]	Between-group difference, P/F units	95% CI	*p*-value
Unadjusted comparison	151/143	333.3 [284.0–401.6]	302.5 [240.0–370.0]	+30.8^a^	-	<0.001
Principal centre-adjusted analysis: study centre	151/143	-	-	+23.8	+8.1–+39.5	0.003
Sensitivity analysis: study centre + preoperative P/F	114/68	-	-	+18.9	+1.9–+35.9	0.030
Sensitivity analysis restricted to site/region strata with both treatment groups	146/63	-	-	+20.7	+3.4–+38.0	0.019

a Unadjusted difference represents the arithmetic difference between group medians and is presented descriptively.

Data availability varied across respiratory outcomes and assessment time points. Outcome-specific denominators and missing observations for each treatment group are reported in Supplementary Table S3.

Before extubation, FiO₂ remained lower in the VibroLUNG group [30.0% [30.0–35.0] vs. 35.0% [30.0–50.0], *p* < 0.001; Figure 3], whereas the unadjusted between-group difference in the P/F ratio was not statistically significant [347.14 [296.5–406.6] vs. 333.81 [273.3–417.1], *p* = 0.351; Figure 2]. After overnight sleep, the P/F ratio was lower in the VibroLUNG group in the unadjusted comparison [319.50 [269.67–390.00] vs. 392.86 [305.00–473.33], *p* < 0.001; Figure 2].

### 3.2 Gas exchange and acid-base status

PaO₂ and PaCO₂ demonstrated statistically significant differences only at selected time points. Early postoperative PaO₂ was comparable between the groups, whereas PaCO₂ was moderately higher in the VibroLUNG group at the early postoperative visit and lower after overnight sleep. The absolute changes were small and did not indicate persistent hypercapnia.

During the early postoperative period, the median pH was lower in the VibroLUNG group [7.34 [7.31–7.37] vs. 7.36 [7.32–7.40], *p* = 0.002], while BE was more negative [−4.20 [−6.20–−2.50] vs. −3.20 [−5.00–−2.10], *p* = 0.007].

### 3.3 Clinical scales

Before surgery, an NRS score of 0 was observed in 151/156 (96.8%) patients in the VibroLUNG group and 135/161 (83.9%) patients in the control group (*p* < 0.001), indicating a baseline imbalance in this measure. The other clinical-scale comparisons before surgery were not statistically significant (Table 4, Figure 4).

**Table 4:** Clinical scales in the early postoperative period.

Time point	Measure	VibroLUNG	Control	OR (95% CI)	p
Before surgery	BPS = 0	154/156 (98.7%)	158/161 (98.1%)	1.46 (0.24–8.87)	1.000
	NRS = 0	151/156 (96.8%)	135/161 (83.9%)	5.82 (2.17–15.57)	<0.001
	RASS = 0	155/156 (99.4%)	160/161 (99.4%)	0.97 (0.06–15.63)	1.000
	GCS = 15	154/156 (98.7%)	160/161 (99.4%)	0.48 (0.04–5.36)	0.618
Early postoperative visit	BPS = 0	94/156 (60.3%)	65/161 (40.4%)	2.24 (1.43–3.51)	<0.001
	NRS ≥4	21/156 (13.5%)	33/161 (20.5%)	0.60 (0.33–1.10)	0.096
	RASS = 0	19/155 (12.3%)	25/161 (15.5%)	0.76 (0.40–1.44)	0.401
	RASS = −1	118/155 (76.1%)	95/161 (59.0%)	2.22 (1.36–3.60)	0.001
	GCS = 15	102/156 (65.4%)	73/161 (45.3%)	2.28 (1.45–3.58)	<0.001
Before extubation	BPS = 0	97/153 (63.4%)	91/160 (56.9%)	1.31 (0.83–2.07)	0.239
	NRS ≥4	27/153 (17.6%)	38/161 (23.6%)	0.69 (0.40–1.21)	0.193
	RASS = 0	129/153 (84.3%)	95/160 (59.4%)	3.68 (2.15–6.30)	<0.001
	RASS = + 1	0/153 (0%)	21/160 (13.1%)	0.02 (0.001–0.35)	<0.001
	GCS = 15	124/153 (81.0%)	114/161 (70.8%)	1.76 (1.04–2.99)	0.034

Values are n/N (%). Odds ratios are presented with 95% confidence intervals. *P* values were calculated using Pearson's *χ*² test or Fisher's exact test, as appropriate. The Haldane–Anscombe correction was applied where a zero cell was present.

**Figure 4:**
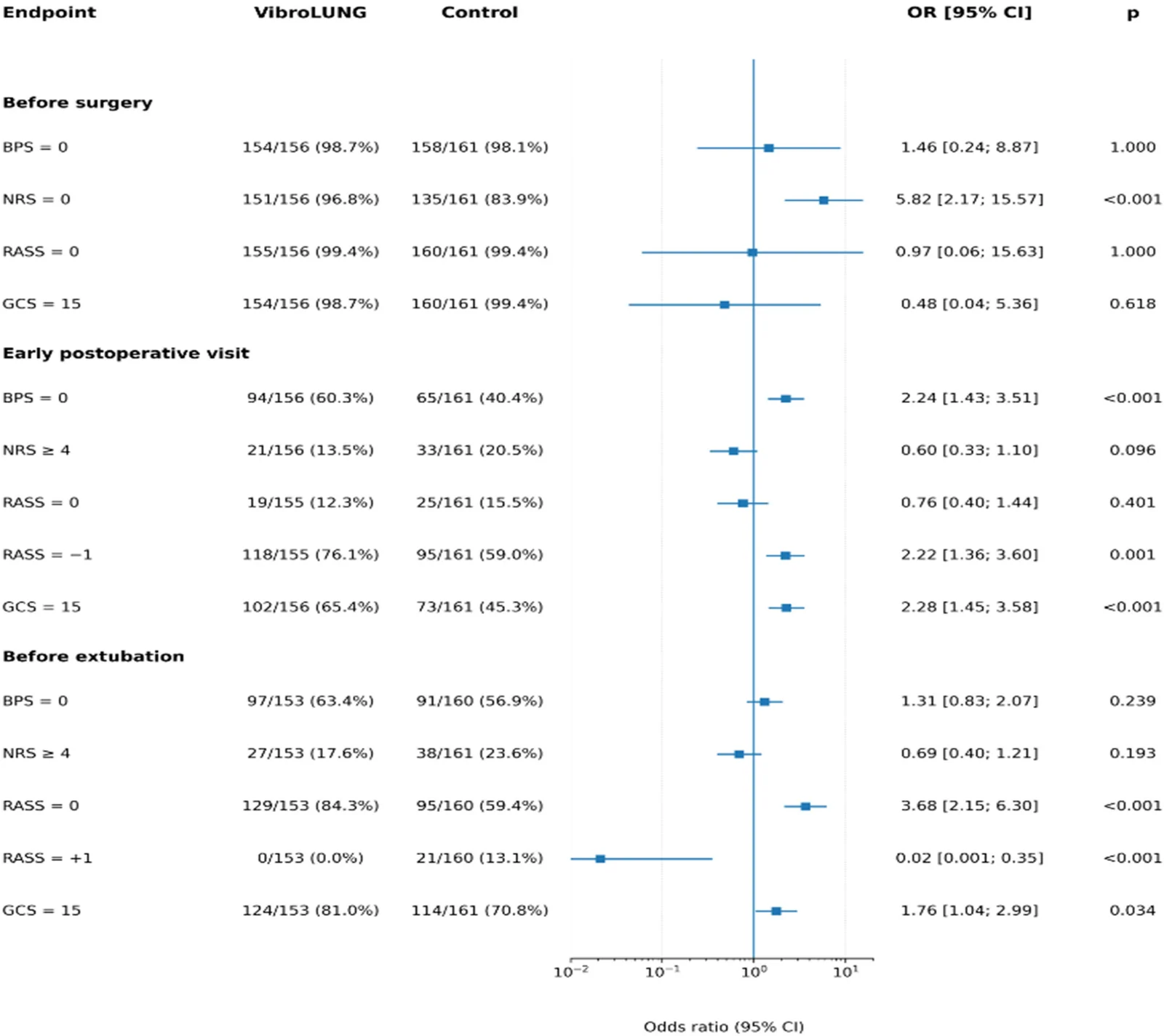
Cross-sectional comparisons of clinical-scale outcomes by treatment group at each assessment time point. Odds ratios are presented with 95% confidence intervals; *p* values were calculated using Pearson's *χ*² test or Fisher's exact test, as appropriate. For odds-ratio estimation in 2 × 2 tables containing a zero cell, the Haldane–Anscombe continuity correction of 0.5 was applied.

At the early postoperative assessment, a BPS score of 0 was observed in 94/156 (60.3%) patients in the VibroLUNG group and 65/161 (40.4%) patients in the control group (*p* < 0.001), while a GCS score of 15 was observed in 102/156 (65.4%) versus 73/161 (45.3%), respectively (*p* < 0.001). RASS = −1 was observed in 118/155 (76.1%) versus 95/161 (59.0%) patients (*p* = 0.001). In contrast, the between-group differences in NRS ≥4 (*p* = 0.096) and RASS = 0 (*p* = 0.401) were not statistically significant.

Before extubation, RASS = 0 was observed in 129/153 (84.3%) patients in the VibroLUNG group and 95/160 (59.4%) patients in the control group (*p* < 0.001), while RASS = + 1 was observed in 0/153 (0%) versus 21/160 (13.1%) patients, respectively (*p* < 0.001). A GCS score of 15 was observed in 124/153 (81.0%) versus 114/161 (70.8%) patients (*p* = 0.034). Differences in BPS = 0 (*p* = 0.239) and NRS ≥4 (*p* = 0.193) were not statistically significant. These clinical-scale findings were considered exploratory given the baseline imbalance and the potential influence of perioperative factors.

### 3.4 Radiological outcomes

Before surgery, the groups were markedly imbalanced with respect to “other clinically significant changes”: 2/156 (1.3%) in the VibroLUNG group versus 41/161 (25.5%) in the control group. Before extubation, this finding was observed in 8/150 (5.3%) versus 26/132 (19.7%), and on postoperative day 3 in 6/156 (3.8%) versus 26/159 (16.4%), respectively. Pneumonia, atelectasis, and pulmonary infiltration were uncommon in both groups (Table 5). Because of the substantial baseline imbalance and the absence of adjustment for baseline radiographic status and centre, these radiographic comparisons were considered exploratory and were not interpreted as evidence of a treatment effect.

**Table 5:** Radiographic findings by group.

Time point	Radiographic finding	VibroLUNG	Control	p
Before surgery	Signs of pneumonia	0/156 (0%)	1/161 (0.6%)	1.000
	Signs of atelectasis	0/156 (0%)	0/161 (0%)	
	Signs of pulmonary infiltration	0/156 (0%)	0/161 (0%)	
	Other clinically significant changes	2/156 (1.3%)	41/161 (25.5%)	<0.001
Before extubation	Signs of pneumonia	1/150 (0.7%)	1/132 (0.8%)	1.000
	Signs of atelectasis	0/150 (0%)	0/132 (0%)	
	Signs of pulmonary infiltration	0/150 (0%)	0/132 (0%)	
	Other clinically significant changes	8/150 (5.3%)	26/132 (19.7%)	<0.001
Postoperative day 3	Signs of pneumonia	0/156 (0%)	2/159 (1.3%)	0.468
	Signs of atelectasis	1/156 (0.6%)	0/159 (0%)	0.495
	Signs of pulmonary infiltration	1/156 (0.6%)	0/159 (0%)	0.495
	Other clinically significant changes	6/156 (3.8%)	26/159 (16.4%)	<0.001

*P* values are unadjusted and were calculated using Pearson's *χ*² test or Fisher's exact test, as appropriate. Radiographic findings were considered exploratory because of the marked baseline imbalance.

### 3.5 Complete blood count

Complete blood count parameters showed postoperative reductions in hemoglobin and hematocrit and an increase in leukocyte count in both groups. An isolated between-group difference was observed for hemoglobin on postoperative day 2 (*p* = 0.022). As these analyses were exploratory, this finding was not interpreted as evidence of a treatment effect (Supplementary Table S1). In the longitudinal ART analysis documented in the clinical study report, significant group-by-time interactions were identified for the complete blood count parameters. These longitudinal findings were considered exploratory and hypothesis-generating.

### 3.6 Coagulation parameters

Coagulation parameters showed descriptive between-group differences across several assessment time points. Importantly, prothrombin time and INR already differed between the groups preoperatively, indicating baseline imbalance. The longitudinal ART analysis documented significant group-by-time interactions for prothrombin time, INR, fibrinogen, and aPTT. However, these outcomes were exploratory and may have been influenced by baseline differences and perioperative treatment. Accordingly, no treatment-specific inference was drawn from these coagulation findings (Supplementary Table S2).

### 3.7 Length of stay

The median ICU length of stay was identical in the VibroLUNG and control groups [2 (2–2) days in both groups, *p* = 0.807; effect size = 0.006]. Specialty-ward length of stay was 15 [11–19.3] days in the VibroLUNG group and 13 [10–18] days in the control group, with a borderline unadjusted between-group difference (*p* = 0.050; effect size = 0.127). After adjustment for region in ordinal logistic regression, allocation to the control group was not an independent predictor of length of stay (OR = 0.674, 95% CI 0.384–1.183, *p* = 0.169), whereas region remained a significant predictor (*χ*² = 145.95, df = 8, *p* < 0.001). The overall model was statistically significant (*χ*² = 150, df = 9, *p* < 0.001; Nagelkerke R² = 0.0829).

### 3.8 Safety

During the study observation period, no adverse events, cases of lack of device effectiveness, device deficiencies, or deaths were recorded.

## 4 Discussion

In this prospective, multicentre, observational, non-randomized study, BARK VibroLUNG use was associated with differences in early postoperative oxygenation measures compared with routine manual percussion chest physiotherapy. The principal finding was a higher early postoperative P/F ratio after adjustment for study centre, accompanied by lower administered FiO₂. This combined evaluation is clinically relevant because the PaO₂/FiO₂ ratio varies with FiO₂ (16), has been evaluated as a prognostic marker after cardiac surgery (17), and SpO₂/FiO₂, although useful as a non-invasive surrogate of PaO₂/FiO₂, has recognized limitations (18–20). This issue is particularly relevant in the early period following cardiac surgery, when pulmonary function may be affected by general anaesthesia, cardiopulmonary bypass, atelectasis, and other perioperative factors (1, 21).

The between-group difference in oxygenation was most apparent during the early postoperative period and was not consistently maintained at subsequent assessments. The attenuation of this difference at later time points may reflect the natural course of postoperative recovery together with changes in oxygen and ventilatory support; however, these factors were not standardized within the study. In addition, the PaO₂/FiO₂ ratio itself varies with FiO₂ (16), and oxygenation indices may also be influenced by respiratory-support conditions (22). Oxygen therapy in the present study was prescribed by the treating physicians according to clinical status and local practice rather than by a standardized study algorithm. Because early postoperative PaO₂ was similar between groups, whereas FiO₂ was lower in the VibroLUNG group, part of the observed difference in the PaO₂/FiO₂ ratio may reflect differences in oxygen-titration practice rather than a direct improvement in pulmonary gas exchange. Accordingly, the early P/F-ratio difference cannot be considered independent evidence of improved gas exchange.

The present findings can be considered alongside previous evidence on postoperative respiratory physiotherapy and oscillatory airway-clearance techniques. Deep-breathing exercises after coronary artery bypass surgery have been associated with reduced atelectasis and improved pulmonary function (7), while high-frequency chest-wall compression or oscillation has been evaluated as a postoperative adjunct after thoracic surgery and pulmonary lobectomy (8, 9). However, the broader evidence on high-frequency chest-wall oscillation encompasses heterogeneous patient populations, clinical settings, and intervention protocols (10, 11). Direct comparison with the present study is therefore limited by differences in surgical populations, devices, treatment protocols, and outcome definitions.

Airway-clearance techniques may facilitate secretion mobilization (23), while lung-recruitment strategies have been investigated for reducing postoperative atelectasis and improving oxygenation after cardiac surgery (24, 25). These concepts provide a plausible physiological framework for an early oxygenation response. However, secretion clearance, regional lung aeration, alveolar recruitment, and ventilation–perfusion matching were not directly measured in the present study. Consequently, these mechanisms remain hypothetical explanations for the observed association rather than demonstrated mechanisms of action of VibroLUNG.

PaO₂ and PaCO₂ did not show a consistent between-group pattern across assessment time points. The transient differences in PaCO₂ were small and did not suggest persistent hypercapnia and may reflect differences in oxygen support, ventilatory management, sedation, hemodynamic status, and other perioperative factors (21). Similarly, the early differences in pH and base excess were exploratory and may reflect the combined effects of surgical stress, cardiopulmonary bypass, tissue perfusion, fluid therapy, and metabolic responses rather than a treatment-specific effect (26). These acid–base findings therefore cannot be attributed directly to VibroLUNG.

Clinical scale findings were supportive rather than primary efficacy outcomes. Their interpretation is limited by the potential effects of pain and analgesia management, sedation, extubation practices, and local patient-management protocols (27–29), as well as by the baseline between-group difference in NRS observed in this cohort. These findings are therefore descriptive and do not provide independent evidence of a VibroLUNG treatment effect.

Radiographic findings were affected by a substantial baseline between-group imbalance in “other clinically significant changes”. Consequently, the subsequent between-group differences cannot provide independent evidence of a VibroLUNG effect without adjustment for baseline radiographic status and centre-level factors. Pneumonia, atelectasis, and pulmonary infiltration were uncommon and did not show a consistent between-group pattern.

Complete blood count findings were exploratory and largely reflected the expected postoperative response to cardiac surgery. The isolated between-group difference in hemoglobin on postoperative day 2 provides insufficient evidence for a treatment-specific effect.

Coagulation parameters were also exploratory and hypothesis-generating. Prothrombin time and INR already differed between the groups preoperatively, indicating baseline imbalance. Interpretation is further limited by the absence of detailed documentation of anticoagulant and antiplatelet therapy and other perioperative factors that may influence coagulation. Taken together, these findings do not support an effect of VibroLUNG on coagulation parameters.

Length of stay provided no evidence of an independent treatment effect. In the adjusted model, region remained significantly associated with length of stay, whereas treatment group did not. Length of stay after cardiac surgery is influenced by multiple clinical factors (30). The borderline unadjusted difference in specialty-ward stay therefore does not demonstrate a treatment-related reduction in hospitalization.

No adverse events, device deficiencies, or deaths were recorded during study participation. These findings describe the observed safety experience in this selected cohort but do not establish the safety of VibroLUNG, particularly for uncommon or longer-term adverse outcomes.

The potential clinical relevance of the present findings lies in the combination of a higher early P/F ratio and lower administered FiO₂. Postextubation oxygen support is an important component of respiratory management in cardiac surgery patients with postoperative hypoxemia, and different oxygen-delivery strategies have been evaluated in this setting (31). However, the between-group difference in oxygenation was not consistently maintained at later assessments, and treatment group was not independently associated with length of stay. The findings therefore represent an early physiological association, rather than evidence of improved overall postoperative recovery or other hard clinical outcomes.

Finally, treatment allocation was non-randomized and reflected device availability and local clinical practice, leaving the possibility of residual centre-level confounding. Differences among centres in oxygen therapy, extubation criteria, sedation and analgesia, perioperative management, and documentation may have contributed to the observed between-group differences.

### 4.1 Study limitations

The principal limitation of this study is the lack of randomization. Patient allocation reflected device availability at participating centers and routine clinical practice rather than random assignment. A second important limitation is the pronounced intercenter and regional heterogeneity. The VibroLUNG group was concentrated predominantly in selected regions, whereas the control group included several regions that were not represented in the VibroLUNG group. Because device availability and local clinical practice influenced treatment allocation, center-specific differences in oxygen therapy, extubation criteria, perioperative management, and documentation may have contributed to the observed between-group differences. Therefore, residual center-level confounding cannot be excluded and should be considered when interpreting the treatment-associated differences. The third limitation concerns the control intervention. Manual percussion physiotherapy was administered according to the local practices of the participating healthcare organizations, and neither its duration nor the technique used was strictly standardized. The fourth limitation is the absence of detailed documentation of concomitant pharmacological therapy, including sedation, analgesia, anticoagulant and antiplatelet treatment, as well as fluid and transfusion management strategies. Additionally, no standardized postoperative respiratory assessment was obtained immediately before the first treatment session; therefore, residual between-group differences in respiratory status at treatment initiation cannot be excluded. The fifth limitation is the baseline imbalance in the radiographic category of “other significant changes”. For this reason, radiographic outcomes cannot be considered independent evidence of treatment effect without additional adjustment. The sixth limitation concerns multiple testing. A large number of physiological, clinical, laboratory, and radiographic outcomes were analysed. Although Holm–Bonferroni correction was applied to *post-hoc* comparisons following significant ART interactions, no global multiplicity adjustment was applied across all exploratory outcome domains. Accordingly, these analyses should be regarded as exploratory rather than confirmatory. The exploratory longitudinal ART analyses did not incorporate study centre as a multilevel effect, and residual centre-level clustering may therefore remain in these outcomes. The seventh limitation is that the investigation primarily reflects the early postoperative period. It does not provide a comprehensive assessment of the effects of VibroLUNG on late complications, rehospitalizations, 30-day outcomes, or long-term recovery. An additional limitation is that the original sample-size calculation was based on a saturation-based effectiveness endpoint rather than specifically on the early postoperative P/F-ratio outcome emphasized in the present manuscript.

## 5 Conclusion

BARK VibroLUNG use was associated with a higher early postoperative P/F ratio after adjustment for study centre and with lower administered FiO₂. These findings do not establish improved pulmonary gas exchange because treatment distribution differed substantially across centres and oxygen management was not standardized. No adverse events, device deficiencies, or deaths were recorded during the study observation period, but these observations do not establish comparative or long-term safety. Confirmation in a randomized trial with centre-stratified randomization, standardized oxygen management, and blinded outcome assessment is warranted.

## Data Availability

The raw data supporting the conclusions of this article will be made available by the authors, without undue reservation.

## References

[1] Badenes R Lozano A Belda FJ. Postoperative pulmonary dysfunction and mechanical ventilation in cardiac surgery. Crit Care Res Pract. (2015) 2015:420513. 10.1155/2015/420513 25705516 PMC4332756

[2] Mello LC Rosatti SF Hortense P. Assessment of pain during rest and during activities in the postoperative period of cardiac surgery. Rev Lat Am Enfermagem. (2014) 22(1):136–43. 10.1590/0104-1169.3115.2391 24553714 PMC4292697

[3] Zencir G Eser I. Effects of cold therapy on pain and breathing exercises among median sternotomy patients. Pain Manag Nurs. (2016) 17(6):401–10. 10.1016/j.pmn.2016.05.006 27746091

[4] Suematsu Y Sato H Ohtsuka T Kotsuka Y Araki S Takamoto S. Predictive risk factors for delayed extubation in patients undergoing coronary artery bypass grafting. Heart Vessels. (2000) 15(5):214–20. 10.1007/s003800070010 11560357

[5] Zayed Y Kheiri B Barbarawi M Rashdan L Gakhal I Ismail E', et al. Effect of oxygenation modalities among patients with postoperative respiratory failure: a pairwise and network meta-analysis of randomized controlled trials. J Intensive Care. (2020) 8:51. 10.1186/s40560-020-00468-x 32690993 PMC7366473

[6] Ahmad AM. Essentials of physiotherapy after thoracic surgery: what physiotherapists need to know. A narrative review. Korean J Thorac Cardiovasc Surg. (2018) 51(5):293–307. 10.5090/kjtcs.2018.51.5.293 30402388 PMC6200172

[7] Westerdahl E Lindmark B Eriksson T Friberg O Hedenstierna G Tenling A. Deep-breathing exercises reduce atelectasis and improve pulmonary function after coronary artery bypass surgery. Chest. (2005) 128(5):3482–8. 10.1378/chest.128.5.3482 16304303

[8] Allan JS Garrity JM Donahue DM. High-frequency chest-wall compression during the 48 h following thoracic surgery. Respir Care. (2009) 54(3):340–3. 10.4187/respcare.09540340 19245727

[9] Park H Park J Woo SY Yi YH Kim K. Effect of high-frequency chest wall oscillation on pulmonary function after pulmonary lobectomy for non-small cell lung cancer. Crit Care Med. (2012) 40(9):2583–9. 10.1097/CCM.0b013e318258fd6d 22732281

[10] Marin A Chiaradia VC Dobre M Brateanu A Baltatu OC Campos LA. High-frequency chest wall oscillation devices: an umbrella review and bibliometric analysis. Comput Biol Med. (2024) 182:109135. 10.1016/j.compbiomed.2024.109135 39288556

[11] Ielo S Carriera L Barone R Mari P-V Coppola A Baglioni S. High-frequency chest wall oscillation in critical care: a systematic review. Respiration. (2026):1–13. 10.1159/000551711 41875299

[12] Sessler CN Gosnell MS Grap MJ Brophy GM O'Neal PV Keane KA. The richmond agitation-sedation scale: validity and reliability in adult intensive care unit patients. Am J Respir Crit Care Med. (2002) 166(10):1338–44. 10.1164/rccm.2107138 12421743

[13] Jain S Iverson LM. Glasgow Coma scale. In: StatPearls. Treasure Island, FL: StatPearls Publishing (2024).

[14] Payen J-F Bru O Bosson J-L Lagrasta A Novel E Deschaux I. Assessing pain in critically ill sedated patients by using a behavioral pain scale. Crit Care Med. (2001) 29(12):2258–63. 10.1097/00003246-200112000-00004 11801819

[15] Van Dijk JF Kappen TH Van Wijck AJ Kalkman CJ Schuurmans MJ. The diagnostic value of the numeric pain rating scale in older postoperative patients. J Clin Nurs. (2012) 21(21-22):3018–24. 10.1111/j.1365-2702.2012.04288.x 23083383

[16] Karbing DS Kjærgaard S Smith BW Espersen K Allerød C Andreassen S. Variation in the PaO2/FiO2 ratio with FiO2: mathematical and experimental description, and clinical relevance. Crit Care. (2007) 11(6):R118. 10.1186/cc6174 17988390 PMC2246207

[17] Esteve F Lopez-Delgado JC Javierre C Skaltsa K Carrio MLL Rodríguez-Castro D. Evaluation of the PaO2/FiO2 ratio after cardiac surgery as a predictor of outcome during hospital stay. BMC Anesthesiol. (2014) 14:83. 10.1186/1471-2253-14-83 25928646 PMC4448284

[18] Carvalho EB Leite TRS Sacramento RFM Nascimento PRL Samary CS Rocco PRM. Rationale and limitations of the SpO2/FiO2 as a possible substitute for PaO2/FiO2 in different preclinical and clinical scenarios. Rev Bras Ter Intensiva. (2022) 34(1):185–96. 10.5935/0103-507X.20220013-en 35674526 PMC9345592

[19] Reddy M Kulkarni M Kanakalakshmi ST Shenoy L KrishnaBhat RR. Non-invasive SpO2/FiO2 ratio (SFR) as surrogate for PaO2/FiO2 ratio (PFR): a scoping review. J Crit Care Med (Targu Mures. (2025) 11(3):221–32. 10.2478/jccm-2025-0024 40765543 PMC12321259

[20] Erlebach R Pale U Beck T Markovic S Seric M David S. Limitations of SpO2/FiO2 ratio for classification and monitoring of acute respiratory distress syndrome: an observational cohort study. Crit Care. (2025) 29(1):82. 10.1186/s13054-025-05317-7 39972458 PMC11837723

[21] Setlers K Jurcenko A Arklina B Zvaigzne L Sabelnikovs O Stradins P. Identifying early risk factors for postoperative pulmonary complications in cardiac surgery patients. Medicina (Kaunas. (2024) 60(9):1398. 10.3390/medicina60091398 39336439 PMC11433804

[22] Vadi SM Sanwalka N Suthar D. Oxygenation index, oxygen saturation index vs PaO2/FiO2×PEEP: a secondary analysis of the OXIVA-CARDS study. Indian J Crit Care Med. (2024) 28(10):917–22. 10.5005/jp-journals-10071-24808 39411293 PMC11471996

[23] Belli S Prince I Savio G Paracchini E Cattaneo D Bianchi M. Airway clearance techniques: the right choice for the right patient. Front Med (Lausanne. (2021) 8:544826. 10.3389/fmed.2021.544826 33634144 PMC7902008

[24] Hu MC Yang YL Chen TT Lee CI Tam KW. Recruitment maneuvers to reduce pulmonary atelectasis after cardiac surgery: a meta-analysis of randomized trials. J Thorac Cardiovasc Surg. (2022) 164(1):171–81.e4. 10.1016/j.jtcvs.2020.10.142 33341273

[25] Martinsson A Houltz E Wallinder A Lindgren S Thorén A. Lung recruitment in the prone position after cardiac surgery: a randomised controlled study. Br J Anaesth. (2021) 126(5):1067–74. 10.1016/j.bja.2020.12.039 33602580

[26] Teixeira FC Fernandes TEL Leal K Ribeiro KRB Dantas DV Dantas RAN. Factors associated with increased lactate levels in cardiac surgeries: scoping review. Rev Bras Enferm. (2024) 77(1):e20230117. 10.1590/0034-7167-2023-0117pt 38511788 PMC10941673

[27] McDonall J Wilson J Botti M Hutchinson A. Patient experience of pain management following cardiac surgery: a mixed methods study. Pain Manag Nurs. (2025) 26(3):271–81. 10.1016/j.pmn.2024.10.012 39542767

[28] SILVETTI S PATERNOSTER G ABELARDO D AJELLO V ALOISIO T BAIOCCHI M. Recommendations for fast-track extubation in adult cardiac surgery patients: a consensus statement. Minerva Anestesiol. (2024) 90(11):957–68. 10.23736/S0375-9393.24.18267-3 39545652

[29] Luo R Fan YY Wang MT Yuan CY Sun YY Huang T. Different extubation protocols for adult cardiac surgery: a systematic review and pairwise and network meta-analysis. BMC Anesthesiol. (2025) 25(1):104. 10.1186/s12871-025-02952-z 40012055 PMC11863475

[30] Zarrizi M Paryad E Khanghah AG Leili EK Faghani H. Predictors of length of stay in intensive care unit after coronary artery bypass grafting: development of a risk scoring system. Braz J Cardiovasc Surg. (2021) 36(1):57–63. 10.21470/1678-9741-2019-0405 33594861 PMC7918390

[31] Theologou S Ischaki E Zakynthinos SG Charitos C Michopanou N Patsatzis S. High flow oxygen therapy at two initial flow settings versus conventional oxygen therapy in cardiac surgery patients with postextubation hypoxemia: a single-center, unblinded, randomized, controlled trial. J Clin Med. (2021) 10(10):2079. 10.3390/jcm10102079 34066244 PMC8151420

