# Supplementary material for: Vibroacoustic lung therapy and early postoperative oxygenation after cardiac surgery: a multicentre prospective study
Source: Front Med (Lausanne). 2026 Sep 17;13:1945462. doi: 10.3389/fmed.2026.1945462 (PMC13657145; doi:10.3389/fmed.2026.1945462)
Supplement: Supplementary file 1 [file Datasheet1.pdf]

Supplementary Material

Table S1. Complete blood count parameters.

Parameter	Visit	VibroLUNG, Med [Q1–Q3]	Control, Med [Q1–Q3]
Erythrocytes	Before surgery	4.66 [4.37–5.00]	4.72 [4.35–5.10]
	After overnight sleep	3.65 [3.30–4.00]	3.62 [3.20–4.12]
Hemoglobin	Before surgery	139.00 [130.00–146.00]	141.00 [130.00–149.00]
	After overnight sleep	109.00 [96.00–117.00]	109.00 [98.00–121.00]
	Day 2	106.00 [93.00–115.00]	108.00 [96.00–121.00]
	Day 3	103.50 [94.00–115.00]	106.00 [96.00–119.00]
	Day 4	105.00 [97.00–117.00]	107.00 [97.00–117.00]
	Day 5	107.50 [97.00–118.00]	110.00 [100.00–118.00]
Leukocytes	Before surgery	7.00 [5.88–8.12]	7.30 [6.40–8.40]
	After overnight sleep	11.60 [9.80–13.40]	12.21 [9.87–14.91]
Hematocrit	Before surgery	41.60 [38.00–44.10]	41.90 [38.75–44.05]
	After overnight sleep	32.20 [29.00–35.70]	32.30 [29.00–36.60]

Table S2. Coagulation parameters

Parameter	Visit	VibroLUNG, Med [Q1–Q3]	Control, Med [Q1–Q3]
Prothrombin time	Preoperatively	16.0 [11.5–19.0]	12.0 [11.3–13.7]
	Early postoperative visit	18.0 [13.5–20.0]	13.7 [13.0–16.0]
	Before extubation	19.0 [13.8–21.0]	14.7 [13.0–18.0]
	After overnight sleep	17.5 [12.7–20.0]	13.7 [12.7–16.0]
INR	Preoperatively	1.1 [1.0–1.3]	1.0 [0.9–1.1]
	Early postoperative visit	1.3 [1.1–1.4]	1.2 [1.1–1.3]
	Before extubation	1.3 [1.1–1.4]	1.2 [1.1–1.3]
	After overnight sleep	1.2 [1.11–1.3]	1.1 [1.1–1.3]
Fibrinogen	Preoperatively	3.4 [2.9–3.8]	3.1 [2.7–3.7]
	Early postoperative visit	3.1 [2.5–3.6]	2.5 [2.1–3.1]
	Before extubation	3.2 [2.7–3.7]	2.9 [2.5–3.4]
	After overnight sleep	3.5 [3.0–3.9]	3.5 [3.0–4.1]
aPTT	Preoperatively	31.0 [27.0–34.4]	31.2 [26.0–35.6]
	Early postoperative visit	35.0 [28.0–42.2]	32.9 [27.0–37.4]
	Before extubation	35.0 [29.8–42.0]	29.9 [24.3–38.0]
	After overnight sleep	33.0 [27.7–40.0]	35.0 [29.0–41.3]

Coagulation outcomes were exploratory and are presented descriptively. No inferential between-group p values are reported because these analyses were not used to assess treatment efficacy.

Table S3. Availability and missingness of key respiratory measurements

Outcome / assessment	VibroLUNG available, n/N (%)	VibroLUNG missing, n (%)	Control available, n/N (%)	Control missing, n (%)
P/F ratio — preoperative	118/156 (75.6%)	38 (24.4%)	75/161 (46.6%)	86 (53.4%)
P/F ratio — early postoperative	151/156 (96.8%)	5 (3.2%)	143/161 (88.8%)	18 (11.2%)
P/F ratio — before extubation	144/156 (92.3%)	12 (7.7%)	150/161 (93.2%)	11 (6.8%)
P/F ratio — after overnight sleep	154/156 (98.7%)	2 (1.3%)	143/161 (88.8%)	18 (11.2%)
P/F ratio — before ICU transfer	120/156 (76.9%)	36 (23.1%)	117/161 (72.7%)	44 (27.3%)
FiO_2 — early postoperative	153/156 (98.1%)	3 (1.9%)	154/161 (95.7%)	7 (4.3%)
PaO_2 — early postoperative	151/156 (96.8%)	5 (3.2%)	144/161 (89.4%)	17 (10.6%)
$PaCO_2$ — early postoperative	151/156 (96.8%)	5 (3.2%)	147/161 (91.3%)	14 (8.7%)
SpO_2 — early postoperative	152/156 (97.4%)	4 (2.6%)	154/161 (95.7%)	7 (4.3%)
pH — early postoperative	156/156 (100%)	0	161/161 (100%)	0
BE — early postoperative	155/156 (99.4%)	1 (0.6%)	161/161 (100%)	0
